# Adapting SBIRT for Batterer Intervention Program groups using motivational approaches

**DOI:** 10.1186/1940-0640-10-S2-O30

**Published:** 2015-09-24

**Authors:** Enid Watson, Carol D Girard

**Affiliations:** 1 Institute for Health & Recovery, USA; 2 Massachusetts Department of Public Health/Bureau of Substance Abuse Services, USA

## Background

To present a public health initiative to adapt SBIRT for Batterer Intervention Programs (BIP) using Motivational Interviewing (MI) approaches.

While studies concur that substance use is not the underlying cause of Interpersonal Violence (IPV), approximately 50% of men in BIP are reported to be using substances at unhealthy levels. The Massachusetts Department of Public Health (MDPH) initiated a pilot to address risky substance use in BIP participants, with the Institute for Health & Recovery (IHR), providing curriculum development, training, and technical assistance.

The curriculum employs an innovative application of SBIRT designed for use in groups, allowing BIP group leaders to provide substance use health education to men in groups. Some studies indicate that MI can result in better engagement with participants than the typical directive, confrontational approaches often used with individuals involved with the criminal justice system. It uses MI strategies adapted to allow the group members to describe the benefits and costs of substance use themselves rather than a more traditional ‘health education’ lecture by a group leader. Validated single item screening tools, for alcohol and for other drugs, were completed in silence during the group, and open-ended questions were asked as the Brief Intervention; leaders were encouraged to ask permission before sharing information in order to give participants a greater sense of control and ownership of their place on the road to change.

## Conclusions

This adaptive SBI approach was deemed successful/very successful by 85% of men (n=215). Group leaders found the material/implementation relevant and useful. Adapting SBIRT for Batterer Intervention Program (BIP) Groups Using Motivational (MI) Approaches is a low-cost, non-threatening initiative to increase ambivalence to change amongst men who batter. Upon implementation in a second program, this curriculum will be used in BIPs across Massachusetts.

## Material and methods

The workshop will include a brief, didactic overview of the curriculum, and then an interactive group experience, using materials and methodology from the curriculum, will be implemented during the session.

## Results

This adaptive SBI approach was deemed successful/very successful by 85% of men (n=215). Group leaders found the material/implementation relevant and useful. Further implementation in the 17 Batterer Intervention Programs across the state will ensue in 2016.
